# A Cloud Infrastructure for Health Monitoring in Emergency Response Scenarios

**DOI:** 10.3390/s24216992

**Published:** 2024-10-30

**Authors:** Alessandro Orro, Gian Angelo Geminiani, Francesco Sicurello, Marcello Modica, Francesco Pegreffi, Luca Neri, Antonio Augello, Matteo Botteghi

**Affiliations:** 1 Institute of Biomedical Technologies CNR, Via Fratelli Cervi, 93, 20054 Segrate, Italy; francesco.sicurello@itb.cnr.it; 2 G&G Technologies Srl, Via Tre Settembre, 99, 47891 Dogana, San Marino; angelo.geminiani@ggtechnologies.sm; 3 LogConsulting, Via Provinciale 175/b, Valsamoggia, 40053 Bologna, Italy; marcello.modica@logconsulting.it; 4 Department of Medicine and Surgery, School of Medicine and Surgery, “Kore” University of Enna, 94100 Enna, Italy; francesco.pegreffi@unikore.it; 5 Unit of Recovery and Functional Rehabilitation, P. Osp. Umberto I, 94100 Enna, Italy; 6 Department of Medicine, Division of Cardiology, Johns Hopkins University, Baltimore, MD 21218, USA; lneri1@jhmi.edu; 7 Department of Medical and Surgical Sciences, University of Bologna, 40138 Bologna, Italy; 8 Accyourate SpA, Via Ulisse Nurzia, 1/A, 67100 L’Aquila, Italy; antonio.augello@accyouratecs.com; 9 Experimental Pathology Research Group, Department of Clinical and Molecular Sciences, Università Politecnica delle Marche, 60121 Ancona, Italy; matteo.botteghi5@unibo.it; 10 Medical Physics Activities Coordination Centre, Alma Mater Studiorum University of Bologna, 40126 Bologna, Italy

**Keywords:** occupational health monitoring, cloud computing, real-time emergency management, wearable device, IoT, digital health

## Abstract

Wearable devices have a significant impact on society, and recent advancements in modern sensor technologies are opening up new possibilities for healthcare applications. Continuous vital sign monitoring using Internet of Things solutions can be a crucial tool for emergency management, reducing risks in rescue operations and ensuring the safety of workers. The massive amounts of data, high network traffic, and computational demands of a typical monitoring application can be challenging to manage with traditional infrastructure. Cloud computing provides a solution with its built-in resilience and elasticity capabilities. This study presents a Cloud-based monitoring architecture for remote vital sign tracking of paramedics and medical workers through the use of a mobile wearable device. The system monitors vital signs such as electrocardiograms and breathing patterns during work sessions, and it is able to manage real-time alarm events to a personnel management center. In this study, 900 paramedics and emergency workers were monitored using wearable devices over a period of 12 months. Data from these devices were collected, processed via Cloud infrastructure, and analyzed to assess the system’s reliability and scalability. The results showed a significant improvement in worker safety and operational efficiency. This study demonstrates the potential of Cloud-based systems and Internet of Things devices in enhancing emergency response efforts.

## 1. Introduction

The rapid development of advanced technologies, such as the Internet of Things (IoT), Cloud computing, and fifth-generation broadband mobile networks (5G), has led to innovations and new opportunities in many fields. In particular, the use of innovative ways to address common medical healthcare challenges is now a reality: the acquisition of a large volume of clinical data, the remote monitoring of a patient at their home or in environments other than the hospital settings, the effective data sharing and collaboration amongst medical centers, the aggregation of (often incomplete and noisy) data and the exploitation of such data in application with strict time and cost constraints, just to cite a few.

Continuous monitoring of vital signs plays a crucial role in modern e-health systems [1], encompassing hospital settings, such as intensive care units [2,3], home care [4,5], and emergency management [6,7,8]. In particular, in occupational settings [9,10], risk reduction can be achieved through the continuous monitoring of stress levels using miniaturized and wearable IoT devices integrated into operators’ clothing, which continuously acquire bio-signals. By leveraging appropriate computing systems, these raw signals can be processed in real time, enabling the development of comprehensive remote monitoring solutions that enhance the safety and well-being of individuals in various environments.

In this context, the integration of IoT technology into emergency management, often referred to as the Internet of Emergency Services (IoES) [8], is particularly significant. This integration aims to enhance the speed and efficiency of responses through real-time data sharing and improved coordination among agencies. Unlike traditional systems that often rely on slow and error-prone manual processes, IoES provides adaptive solutions capable of responding to the increasing frequency and complexity of emergencies, such as natural disasters and pandemics, which demand quick and efficient actions to manage rapidly changing conditions.

**Figure 1:**
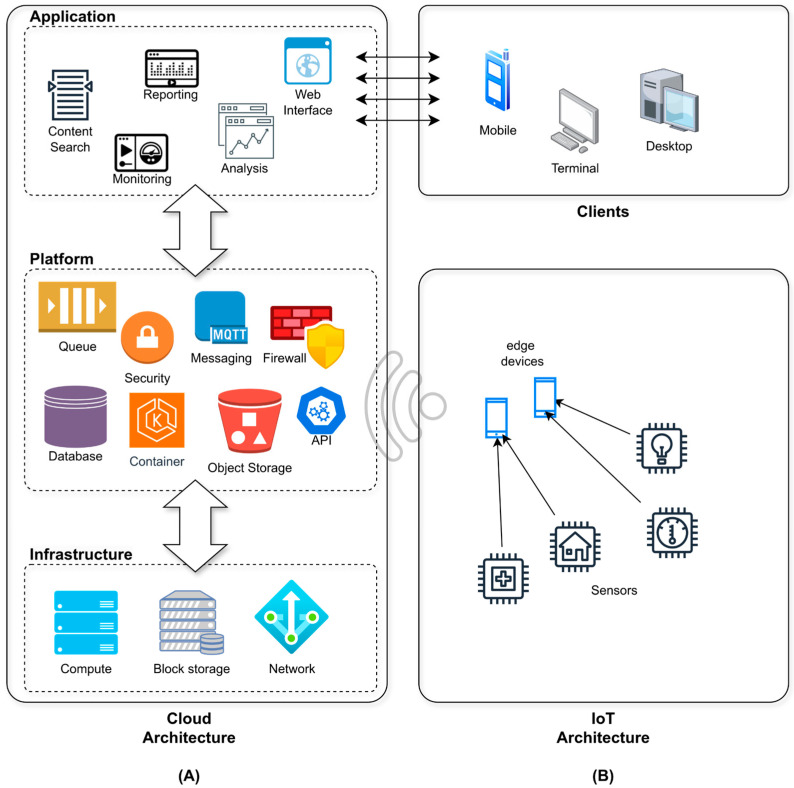
This diagram illustrates the typical Cloud (**A**) and IoT (**B**) architectures along with their respective ecosystems. It depicts the data flow and interactions among various components, including mobile devices, terminals, and containerized services. The diagram highlights the integration of IoT devices and communication protocols, such as MQTT, with Cloud-based infrastructure, emphasizing how these elements collaborate to enhance operational efficiency and responsiveness in data processing and analysis.

Cloud computing architectures, by offering resources as sets of virtualized functionalities, provide the necessary storage and processing capabilities, allowing data gathered by devices to be processed promptly. On the other hand, IoT architectures, typically composed of sensors, edge devices, and actuators, are designed to capture and transmit environmental or physiological data in real time. IoT components, while effective in collecting data from the environment, often lack the computational power to process them independently and could have energy limitations. However, in emergency management, where real-time responsiveness is paramount, relying solely on Cloud-based solutions may introduce latency issues due to network delays. Therefore, a multi-tier architecture is required, where edge computing nodes, fog computing layers, and the Cloud work in tandem [11,12,13,14]. Figure 1 illustrates the architecture of a Cloud-based IoT application.

Despite extensive research in the field of e-health monitoring, a lack of versatile solutions or generic frameworks that can be easily adapted to meet specific requirements currently remains. The challenges in developing a comprehensive healthcare monitoring system include time and budget constraints, difficulties in integrating various hardware and software technologies, and the need to integrate solutions with existing ICT systems, such as authentication and role management systems, as well as databases containing clinical data. This ensures seamless data flow and coherent user and access management, ultimately enhancing the system’s overall effectiveness. Where the use of wearable devices is a crucial factor, the availability of truly comfortable and functional solutions remains very limited.

The integration of wearable IoT technology into healthcare services that use digital and information technologies to improve health management and patient care (e-healthcare services) has gained increasing attention, as demonstrated by constant growth in worldwide revenue for the IoT market [15] and the extensive scientific literature [16,17,18,19,20]. The general architecture of IoT in e-healthcare comprises five connected components: (1) sensor devices that gather data from the environment or patient; (2) networking for transmitting data via networks such as Wi-Fi or cellular; (3) edge devices that perform local data processing; (4) ICT platform for collecting, storing, and processing data from multiple sources (including relational and non-relational databases); and (5) application interfaces for healthcare providers and patients to access data and make informed decisions.

In the context of this study, the infrastructure proposed in [21] aims to optimize large-scale data acquisition from a heterogeneous set of IoT medical sensors (including activity, blood pressure, temperature, heart and breath rate), with a primary focus on real-time emergency response. A prototype system for monitoring oxygen saturation (SpO2) and electrocardiogram (ECG), and for alerting in the waiting area of an emergency room, was proposed in [22]. It was specifically developed to manage overcrowded emergency departments where large numbers of unattended patients need to be remotely monitored to assess the progression of health conditions. The work presented in [23] aims at implementing an infrastructure for patient localization and tracking, based on a Received Signal Strength (RSS) method, with the goal of classifying movements and detecting hazardous situations. Other studies, such as [24,25], explore the usability and feasibility of a system that combines Bluetooth (BT) and Near-Field Communication (NFC) technologies to measure parameters, such as blood pressure, body weight, glucose, oxygen saturation, and ECG signals, whereas ref. [26] focuses on the remote monitoring of elderly patients (temperature, heart beat, muscle activity, and ECG) using ZigBee-enabled devices.

On the other hand, a significant part of the literature has focused on architecture solutions. In particular, ref. [27] highlights the importance of the message-broker pattern for decoupling data producers and consumers, thereby enabling the use of distributed computing. Additionally, ref. [28] demonstrates how an IoT system can be constructed using cost-effective devices and incorporating teleconsulting services in the pipeline to facilitate doctor collaboration. Furthermore, ref. [29] proposes a solution in which data are collected by a gateway through a low-power Wide-Area Network (WAN) that also incorporates alarming functionality on the edge devices. In ref. [30], the IoT devices are equipped with Wi-Fi connectivity so they can work independently on a mobile gateway. An important class of proposed solutions focuses on architectural aspects [31,32] and, in particular, Cloud computing [33,34]. For instance, ref. [35] focuses on monitoring Mild Cognitive Impairments (MCIs) and Chronic Obstructive Pulmonary Disease (COPD), while ref. [36] addresses the monitoring of vital signs obtained from printed wearable devices and remote data processing. Additionally, ref. [37] proposes a Cloud solution for fall detection.

The cited references generally offer effective solutions for the specific problems described and suggest architectural patterns for developing new ones. However, neither the solutions cited nor those available on the market appear to be generic enough or readily available as open-source options that can be utilized in other applications with minimal customization. Furthermore, to the best of our knowledge, no solutions currently exist that integrate wearable devices with robust Cloud infrastructure while ensuring data reliability and maintaining the same satisfactory characteristics

In this study, we describe a project developed in collaboration with the Italian National Red Cross Society (Croce Rossa Italiana, CRI), aimed at monitoring the health status of personnel involved in rescue operations. As part of this project, a large-scale Cloud application has been developed to enable real-time monitoring of vital signs using Internet of Things (IoT) technologies. The focus of this study is to explore the potential of IoT technologies for monitoring vital signs in a large-scale setting, as well as the role of Cloud technologies in managing large-scale monitoring applications. Moreover, an innovative, high-comfort smart shirt equipped with medical device-level sensors will be introduced, serving as the primary IoT component of the Cloud application. This shirt will enable the application to continuously monitor the vital signs of rescue personnel, including temperature, BPM, ECG, respiratory traces, and fall detection, providing alerts if any deviations from normal values are detected. This can help ensure safety and well-being while enhancing the overall efficiency of rescue operations.

## 2. Materials and Methods

### 2.1. Research Methodology

**Figure 2:**
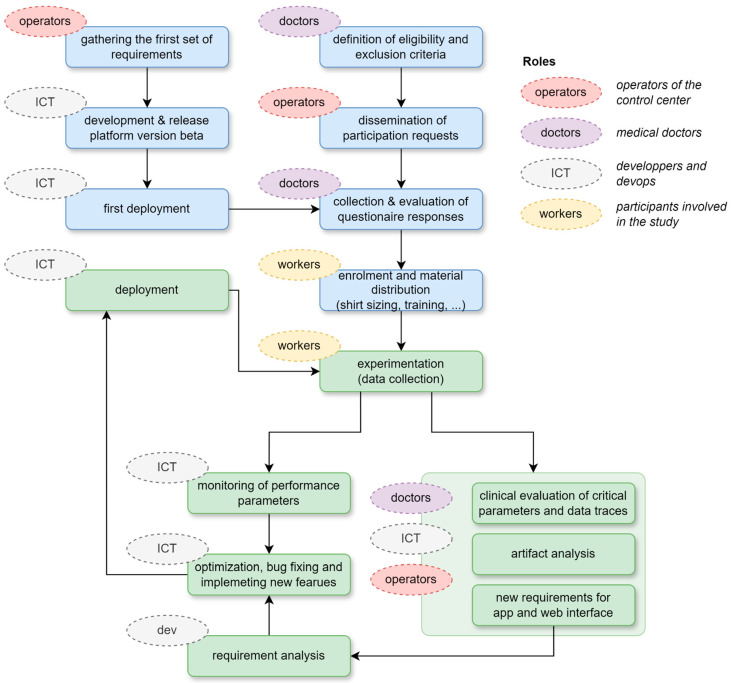
This diagram outlines the step-by-step process followed in the study, starting from the release of the first beta version of the platform and the collection and evaluation of questionnaires for participants’ enrolment. It includes key phases such as participant eligibility screening, monitoring sessions, data collection, and artifact analysis. The workflow also highlights the data validation, analysis, and assessment of the system’s performance and reliability under real-world conditions. For each step, the main categories of roles involved are highlighted: CRI personnel (control center operators, medical doctors and participants) and ICT team.

The research methodology for this study is outlined in Figure 2 and includes two initial parallel steps: (1) developing the first beta version of the platform based on preliminary requirements, and (2) conducting the enrolment process, which begins with defining eligibility criteria, followed by dissemination through the central and local channels of the CRI to attract potential participants, and setting up the experimentation. The experimentation followed an iterative process, where the outcomes of monitoring and user experience were used to generate new requirements and incrementally improve the platform in terms of efficiency and reliability, user experience of the web interface and smartphone application, and accuracy of alert generation by enhancing artifact and false-positive detection.

In particular, initial requirement analysis allowed us to identify several application-specific requirements, including the following: (1) a website that allows all personnel involved to access data within a security schema (ACL-like) that specifies which users are granted access to each type of information; (2) integration with the existing authentication system via OpenID; (3) an onboarding tool for the rapid registration of volunteers interested in the study, including the acquisition of necessary information such as GDPR consent; (4) management of clinical and anamnesis information and to automatically evaluate general eligibility over time; (5) IoT wearable devices for monitoring vital signs along with related ICT components for online and offline use of acquired data; (6) real-time analysis of vital signs to identify changes in health conditions that may lead to potential risks and the generation of suitable alert events; (7) management of the alarm workflow, including involvement of medical staff, assignment alarms to appropriate specialists, and closing of resolved or false-positive alarms.

These requirements are well suited to be addressed with a Microservice Cloud computing Architecture. The Cloud is a new way of delivering computing resources, such as storage and processing power, over the internet in a distributed way. The architecture of Cloud-based systems is constantly evolving to meet the needs of these systems, and one popular approach is the use of microservices. A microservices architecture involves breaking down a large, complex system into smaller, independent components that can be developed, tested, and deployed separately. This can make it easier to scale and update the system, as well as make it more resilient to failure [38,39].

### 2.2. Cloud Architecture

**Figure 3:**
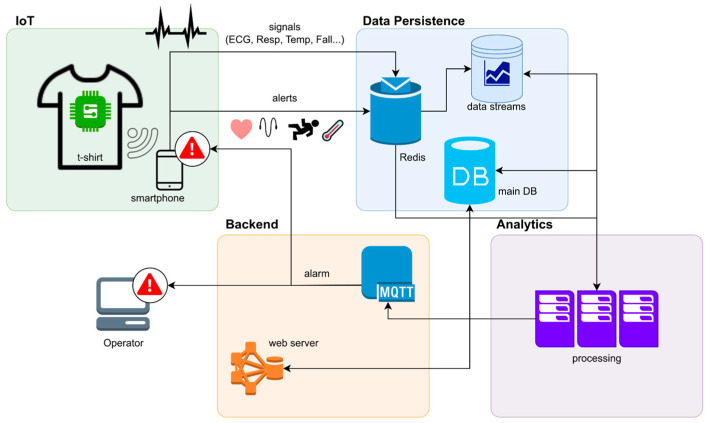
Architecture and main components of the proposed solution: real-time data are collected from the wearable device, which communicates with a smartphone to provide immediate alerts regarding health parameters such as heart rate, respiratory rate, temperature, and fall detection. The data flows into a robust Data Persistence Layer that employs both Redis for fast data caching and a main database for long-term storage. The backend system utilizes the MQTT protocol to effectively manage alarm notifications, ensuring timely responses to critical health events. Finally, the analytics component processes the collected data, enabling comprehensive data analysis.

In this paragraph, we describe the general Cloud architecture of the proposed solution, with subsequent paragraphs delving deeper into each component. The architecture schema (Figure 3) illustrates the main components of the system, their interactions, and the data flow generated by IoT devices. These components are divided into four main layers: (1) The IoT layer, which includes the hardware components (the device sensor printed onto a t-shirt) and the software components that directly interact with them. This primarily consists of a Message Queuing Telemetry Transport (MQTT) microservice that receives IoT measures generated from the devices. (2) The persistence layer, which consists of a Structured Query Language (SQL) database for managing the web application, alongside several NoSQL databases that store IoT measures and alarms. (3) The web frontend and backend layer, which exposes a website for end users to access the application and includes a set of Application Programming Interfaces (APIs) for automating specific procedures (such as onboarding, device assignment to users, and data export), as explained later. (4) The analytics layer, which is responsible for filtering incoming data streams, ensuring data quality, processing raw data, and generating real-time alarms based on predefined criteria.

Each layer comprises several interacting services, all secured with authentication and SSL communication. Additionally, all services are deployed in an elastic environment, referring to the system’s ability for the automatic provision or deprovision of resources in response to workload changes throughout its lifecycle. This feature is crucial for achieving cost reduction, reliability, and prompting response times. Table 1 displays implementation details for each service in the platform.

Data generated by the sensor worn by the participant (IoT layer) are initially sent to the smartphone, from where they are transmitted to the Cloud platform (backend layer) via the MQTT protocol. The message is then permanently stored in the central database (persistence layer) and simultaneously sent through a Lambda function that filters the signal based on predefined thresholds to determine if it represents a potential danger (alert). If it does, the data are forwarded to the analytics module (analytics layer), which assesses whether the signal is a true or false positive. For example, a false positive could be an anomalous spike in temperature. If the signal is classified as true, an alarm is generated and propagated to the clients (web clients used by operators in the control center) and to the smartphone of the person wearing the device.

**Figure 4:**
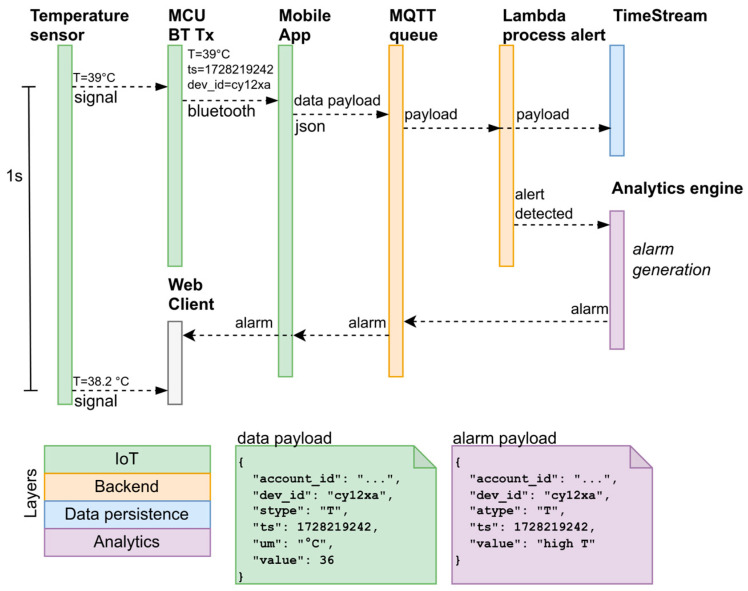
Sequence diagram describing the reading of a signal from the device (e.g., a temperature sample at 1/300 Hz, corresponding to a 5 min interval), the storage of the data in the Cloud system, the generation of an alarm, and the propagation of the alarm to the mobile app and the web client.

The main data flows of the application, primarily concerning real-time alarm management and the procedures for acquiring data from the devices, are described in Figure 4, using temperature as an example. The management of other parameters from the sensors follows a similar workflow: two Lambda functions process the ECG and respiratory traces (at 250 Hz), generating average frequency values (BPM and Resp Frequency) at 1/60 Hz. The subsequent analysis of alerts and alarms is based on these calculated values. Fall detection analysis is performed at the edge, so the alarm message is sent to the platform without any preprocessing. In contrast, motion analysis, which requires downstream data processing, is performed on the Cloud platform.

### 2.3. IoT Layer

The IoT layer includes both hardware (HW) and software (SW) components for continuous multiparameter monitoring. The YouCare medical wearable device (Accyourate wearable technology [40]) consists of textile garments equipped with innovative non-invasive polymeric sensors embedded in the clothing via ink-jet printing. These devices’ wearability and ergonomic characteristics enable a novel method of detecting biovital and kinetic parameters, providing dynamic, real-time measurements of an individual’s status. The device, which is a type IIa-certified medical device, resembles a T-shirt that covers the upper chest. It has been used to monitor several biovital parameters in home settings and during everyday activities, across different contexts, such as healthcare, workplaces, sports, and scientific research. The parameters measured and calculated included heart rate (HR), respiratory rate (RR), skin temperature (SkT), and heart rate variability (HRV, expressed as time–domain and frequency–domain parameters) [41,42]. The sensors within the device, which offer comfortable wearability and favorable ergonomic features, also detect kinetic parameters, enabling dynamic, real-time measurements. Sensor signals are digitized and processed by a miniaturized wearable control unit, which records the data and transmits them to a smartphone app via Bluetooth. The control unit is equipped with various sensors, including a BLE module based on SoC NORDIC nRF52811, Bluetooth 5.1 Low Energy, ECG (1 channel, 16-bit resolution), respiration monitoring (1 channel bioimpedance, 20-bit resolution), temperature, accelerometer, gyroscope, shock detection, 8 MB memory, RGB LED, Micro-USB charge, 3.7 V 190 mAh battery, autonomy > 30 h, 9.5 g weight, 250 Hz sampling frequency (ECG, respiration and accelerometer) and 1/300 Hz (temperature), and two-channel analog front end (24-bit delta-sigma converter). The device’s quality was previously assessed in a study [43] that compared 7200 ECG signals from 20 patients with those acquired from a commercial ECG monitor in a controlled clinical setting.

The textile garment is composed of 90% polyester and 10% elastane, with the wearable device weighing approximately 80.8 g. All validations of the sensor and the wearable device were conducted under dry skin conditions. We experimentally found that increased humidity—whether from sweating or environmental factors—tends to elevate the amplitude (in volts) of the signal without compromising its quality or stability during movement. To maintain the amplitude of the signal within the operational range of the signal acquisition system, we developed an auto-scaling mechanism specifically designed to normalize the signal and prevent saturation of the digitizer inputs. This ensures that the analysis is accurate under all operating conditions.

**Figure 5:**
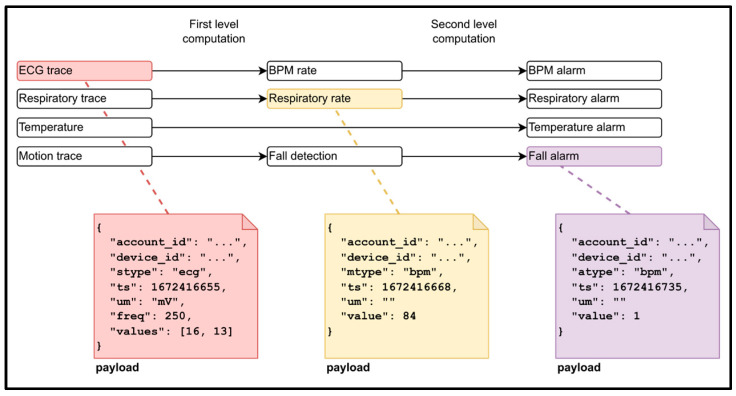
The diagram illustrates the data flow from raw to processed data, showcasing the payload format along with examples. Each payload includes identifiers for both the account and the device, the timestamp of the measurement or event, and corresponding values that vary based on the type of device.

Each sensor detects raw parameters, which are processed to generate derived parameters following the data flow outlined in Figure 5. Depending on system configuration and internet connectivity stability, derived parameters can be computed remotely in the Cloud (Cloud), locally on the smartphone CPU (edge), or through a hybrid approach. Raw parameters include ECG traces, respiratory traces, body temperature, and motion data. The first level of derived parameters includes BPM (heart rate), RespRate (respiratory rate), fall detection (frontal, lateral, and posterior), and shock detection, all computed from raw signals. The second level involves the generation of alert events to identify anomalies or abnormal health conditions.

This process facilitates real-time data exchange with the Cloud platform, where the post-processing and analysis of collected data occur using dedicated, proprietary software and algorithms. Raw data, except for specific cases delegated to the smartphone (e.g., skin temperature conversion, fall detection, and so on), are processed in the Cloud. Artifacts are identified using the algorithm in [43] and subsequently discarded. Derived HRV time–domain parameters include RMSSD, standard deviation (SD) of all NN intervals (SDNN), SD of R-R intervals (SDRR), RMSSD to MeanNNI ratio (CVSD), percentage of successive R-R intervals differing by more than 20 ms (pNNI-20) or 50 ms (pNNI-50), SD of successive differences between NN (SDSD), Mean of NN (M-NNI), and SDNN divided by MeanNN (CVNNI). Derived HRV frequency–domain parameters include low-frequency power (LF), high-frequency power (HF), LF/HF ratio, normalized LF power (LFnu), normalized high-frequency power (HFnu), and total spectral power.

**Figure 6:**
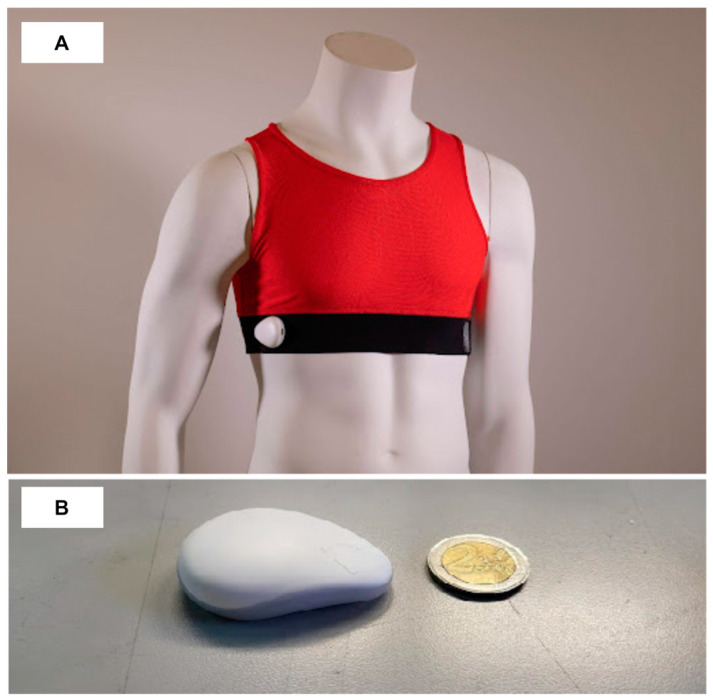
The IoT device comprises a sensor-equipped t-shirt (**A**) and a data collection and preprocessing unit (**B**). This unit gathers and transmits data to a mobile smartphone application via Bluetooth, which then forwards the information to the Cloud platform.

The clothing designer assigned sensorized textiles to subjects, ensuring a specific fit to each breast circumference. Each subject received an experimental kit consisting of a YouCare T-shirt (Figure 6A), a control unit (Figure 6B) with an anonymous serial number, and accessories, including a battery charger, USB type C cable, and the YouCare app to be installed on the users’ personal smartphone. Each medical device was paired with a control unit using an individual pseudo-anonymized code known only to the scientific staff.

The smartphone app offers typical features for this kind of application: device registration on the platform upon initial access, Bluetooth pairing with the Micro Controller Unit (MCU), authentication, initiation and termination of monitoring sessions, and data visualization.

Before transmission to the Cloud platform, sensor datasets are enriched with metadata (user identifier, device identifier, and timestamp), packed in JavaScript Object Notation (JSON) format, and compressed. The data are transmitted using the MQTT protocol, where clients publish messages to specific topics, and the broker manages distribution to subscribers. This decoupling of the sender and receiver allows for independent message transmission, simplifying the overall architecture’s scalability. The MQTT broker, a component of the IoT layer, also communicates with the persistence layer by forwarding sensor data to a NoSQL database and sending MQTT events to an SQS queue, which acts as a buffer to send messages in batches and prevent overloading the system for real-time processing by the Cloud platform. More details are provided in the persistence layer section.

### 2.4. Data Persistence Layer

The Data Persistence Layer (DPL) consists of components and software interfaces that encapsulate routines for accessing data that need to be permanently stored in the Cloud platform. The two primary data flows feeding the DPL are from IoT devices through the IoT Layer and from the Web User Interface. The first data flow includes streams of ECG and respiratory traces, processed time series (such as BPM and temperature), generated alerts, and messages tracking the connectivity status of the devices. Due to the nature of this information (key/value or unstructured stream format), NoSQL databases were chosen: TimeStream for storing data streams and time-series measures, and Redis for storing device status. NoSQL databases offer several advantages over traditional relational databases in this context: they are designed to automatically scale horizontally (by adding new computational nodes) as the amount of data grows; they are well suited for applications requiring high scalability and availability, and they provide efficient access to large volumes of data.

The second data flow originates from user interactions with the Web Interface for managing clinical and anamnesis data, logistic information about device provision, and the current status and history of active alarms. These data must be managed with respect to authorization and permissions (ACL), using the basic Create, Read, Update, and Delete (CRUD) operations. For this purpose, a relational database (PostgreSQL) was selected, as it provides a static, clear, and well-defined data structure that maps seamlessly to application domain objects.

A set of Lambda functions are also part of this layer. In the context of Cloud computing, the Lambda function is a flexible way to provide event-driven computing without taking care of provisioning the underline computational resources. They can be easily connected to input events (for example, MQTT or SQS events), and their output can trigger another event, making it possible to implement a fully managed data workflow. In the proposed platform, the Lambda functions are used mainly to preprocess the input coming from the IoT Layer and to generate the alarm:

1. The computation of BPM rate, respiratory rate, and motion-related events;
2. The generation of alarm to be shown in the web user interface;
3. Updating the relational database, which contains the status of the devices.

As said before, depending on the configuration, all computations performed by Lambda functions can be also performed directly by the mobile device (Edge). Finally, Simple Queue Service (SQS) components are used to buffer the input of Lambda functions in order to aggregate the data in batch and reduce the amount of calls.

### 2.5. Web Backend/Frontend Layer

The user interface and associated business logic are implemented in the final layer of the system’s architecture. This layer includes a web backend built using the Flask microframework, which provides an API for the web frontend. The backend microservice implements OpenID+JWT-based authentication and interacts with both SQL and NoSQL databases to retrieve data for the user interface.

The backend system has been deployed on an elastic load balancer, which automatically distributes incoming traffic across a group of servers. This helps to ensure that the backend can handle a high volume of traffic and can scale up or down as needed to meet changing demand. The load balancer routes incoming requests to a pool of worker servers, which are responsible for processing the requests and returning the appropriate responses. The number of worker servers can vary over time, depending on the workload, allowing the system to adjust its capacity as needed to handle the load.

The topmost component of the system is the web interface, which is implemented using the Vue open-source JavaScript framework as a single-page application (SPA). It is based on the model-view-viewmodel (MVVM) paradigm, which separates the application’s data model from its presentation layer and allows developers to build complex, interactive user interfaces more easily. The web interface allows one to perform all operations necessary to view and modify data and to monitor the IoT parameters with plots and forms.

One of the main features of the interface is to open and maintain, during the web sessions, a stable connection with the Data Persistence Layer through an MQTT-based websocket client. The client subscribes to the topics necessary to keep the interface up to date in response to new IoT events. In particular, it is used to show the status of the devices (connected/disconnected) and to receive alarms in real time.

### 2.6. Analytics Layer

This layer undertakes the tasks of processing incoming data streams, implementing several algorithms of filtering, artifact detection and QRS and BPM computation. Identifying artifacts poses a critical and recurring challenge in IoT systems, given the typical deployment of IoT devices in uncontrolled environments. The main causes include poor device placement, interference issues, and anomalous body movements that could interfere with the acquired signal.

ECG analysis: The ECG analysis is performed with methods specifically developed for the device in use; filtering and artifact detection is performed with an algorithm [44] that leverages the distinct measurable statistics in good signals compared to artifacts within a 2 s window. ECG signals are then processed to extract the QRS with a variant [41] of the well-known Pan-Tompkins method [45]; finally, the BPM is evaluated as an average of the number of peaks over a 5 s window.

Temperature: The values received at 1 Hz from the central unit are cached and sent to the Cloud every 5 min. These values are not subjected to specific analysis before sending to the Cloud platform

Respiratory Trace: The analysis of Respiratory Trace is analyzed with a butterworth low-pass filter (order size 4, cut off 4 Hz) and averaged with a moving window of 25 samples to reduce noise. The frequency is then computed as a count of peaks every 60 s.

Fall detection: Body movements are measured with an accelerometer device incorporated into the central unit, and fall is recognized when a sudden acceleration of more than 6 g is detected.

Alarms: One of its pivotal functions on the analytics layer is the generation of real-time alarms, triggered in response to detected anomalies or critical events, thus ensuring timely interventions and proactive management. To mitigate the risk of false positives and enhance alarm accuracy, the layer employs a window mean filtering and a mechanism that stops the generation of new alarms if an alarm of the same type is already in open state.

### 2.7. Case Study

The presented platform has been developed to monitor the vital signs of a group of paramedics and medical workers employed by the Italian National Red Cross Society (Croce Rossa Italiana, CRI) during their work session. The invitation to participate was voluntary, following the signing of informed consent forms, and was extended to all CRI national committees of the organization. Compilation of a questionnaire enabled the selection of volunteers based on specific acceptance criteria, which included the following: absence of heart disease, chronic cardiovascular diseases, motor neuron diseases, severe motor disabilities (such as paraplegia), chronic diseases with progressive effects; legal age (greater than 18 years); weight and height within a healthy range (neither severely underweight nor overweight); and technical specifications regarding the smartphone. Furthermore, medical staff thoroughly evaluated the presence of critical medical history, past interventions, or hospitalizations before confirming inclusion in the experimentation and, when necessary, consulted with the medical specialists on our team for the final decision on eligibility.

Finally, each participant received an IoT device, consisting of the sensor-equipped shirt. Additionally, participants will undergo several hours of training on correctly wearing the shirt and utilizing the accompanying mobile application.

According to GDPR, only a minimal set of information is included (see Table 2). Moreover, data are classified in different categories with their own set of permissions based on use. For example, access to clinical information is granted only to medical staff upon justified request or for the management of a critical alarm. Information related to alarm and technical measures is accessible only to operations room staff.

To enhance security measures, OAuth2 and OpenID are employed for authentication, delegating authentication and permission management to the GAIA (Gestione Avanzata ed Integrata dell’Anagrafica) platform of Italian Red Cross [46].

Out of the 3600 invited, only about 2935 participants passed the selection criteria, were recruited, and began the experiment after a few weeks. During the study, some participants were excluded for various reasons (personal reasons or errors in parameter recording and so on), leaving 892 who actively participated in the project.

**Figure 7:**
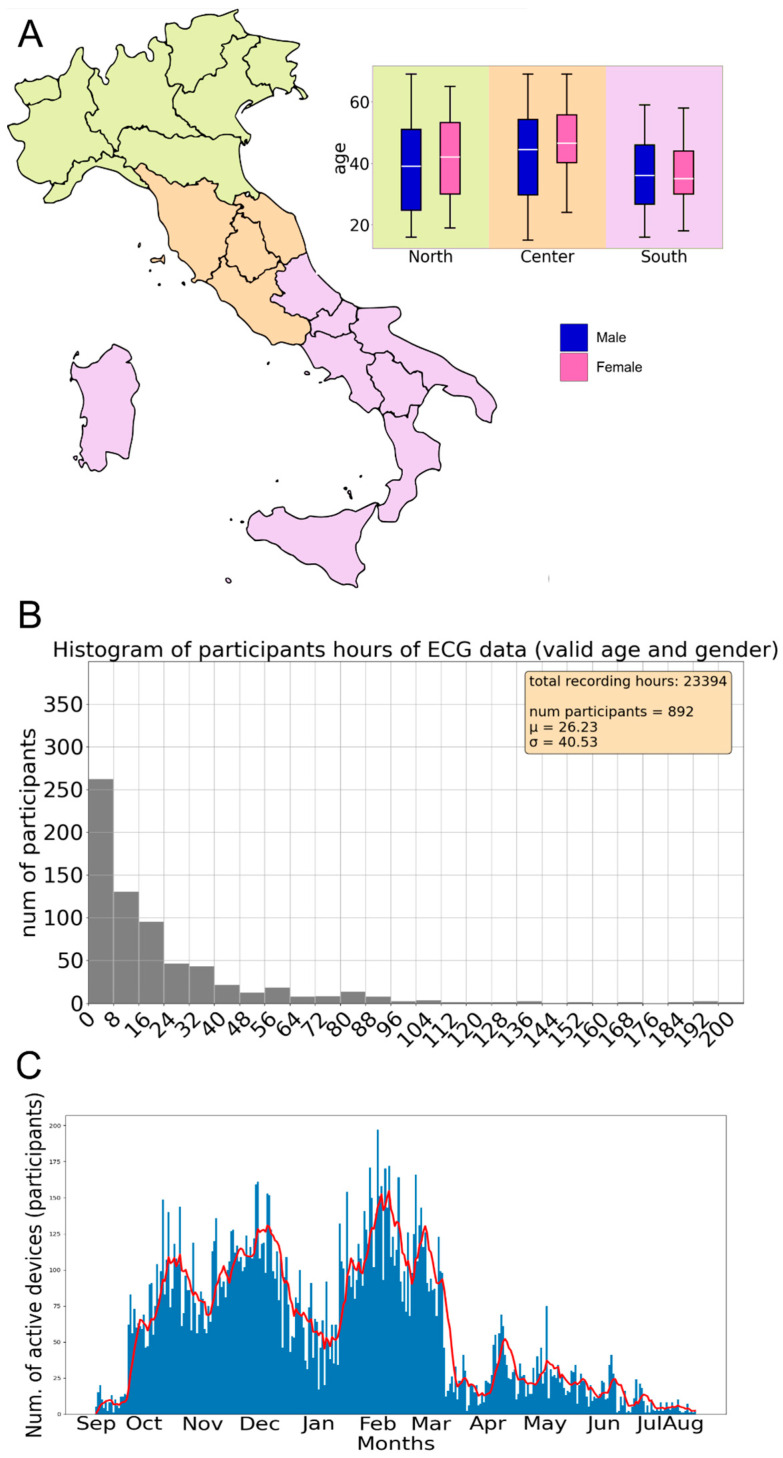
(**A**) Boxplots of the statistical distribution of age, divided into Male (blue) and Female (pink), and into the three macroregions where the project took place: North (green), Center (orange), and South (violet) of Italy. Each boxplot bar represents the statistical distribution of the corresponding dataset, including the mean (white line), the 68th percentile (the border of the box), and the max/min values (the extreme lines) (**B**) histogram distribution of recording hours per participant (device). Each participant recorded an average of 26.23 h for a total of 23,394 h over about 1 year of experimentation; (**C**) distribution of the number of active devices over the duration of experimentation.

The participants are fairly evenly distributed between males (52%) and females (48%), have an average age of 41.5 ± 14.3 years (40.3 ± 14.9 for males and 43.0 ± 13.33 for females), and are across the entire Italian territory, organized by regional committees, and grouped into northern, central, and southern regions (see Figure 7).

This study lasted approximately one year (330 days with 316 days with at least 1 active device), totaling about 23,000 h of activity, where 1 h counted for each participant if at least 1 stream signal (ECG or Respiratory Trace) was registered in the platform (see Figure 7 for more information).

In this experimentation, a total of 100 GB of persistent data was collected from the wearable devices deployed in the monitoring system, with ECG data being the most prominent. The data collection process involved capturing a wide range of vital signs, including ECG and breathing patterns, during various work sessions. The ECG data underwent a rigorous validation process to ensure their quality and reliability. All artifacts and noise were removed using the proprietary methods described previously, before performing any further analysis. This step was crucial for obtaining accurate intermediate measurements and, ultimately, for generating alarms. Additionally, the dataset was assessed from a clinical perspective, with a particular focus on cinematic and cardiorespiratory data.

A total of about 4000 alerts (see Table 3) were generated throughout the monitoring period, and more than 72% were related to BPM anomalies, with 15% related to body temperature increasing above the risk threshold. Among these, more than 40% were identified as false positives (artifacts or incorrect wearing of the device). This high number of false positives underscores the importance of continuous refinement of our alerting system. We recognize the need for improvements to reduce the incidence of false alerts and enhance the precision of the alerting mechanism, ensuring that only real issues are flagged in order to ensure accurate evaluation of the risk in working activity.

Looking ahead, we plan to implement a comprehensive clinical labeling process for the ECG data. This process will involve categorizing different types of ECG events and correlating these events with the clinical data collected. Our goal is to conduct an in-depth correlation analysis between the clinical data and the alerts received. This analysis will help us to better understand the relationship between vital sign patterns and real-time alerts, contributing to the refinement of our monitoring platform. Our future work aims to improve the predictive capabilities of our system by enhancing its accuracy and reliability. By doing so, we hope to not only improve emergency response but also gain valuable insights into vital sign trends and their connection to alert events. This ongoing research will play a crucial role in advancing our understanding of how wearable technology can be optimized for effective health monitoring and emergency management.

## 3. Results

In this section, we describe the web interface of the proposed application, its main workflows, and the performance and scalability analysis.

### 3.1. Web Interface

**Figure 8:**
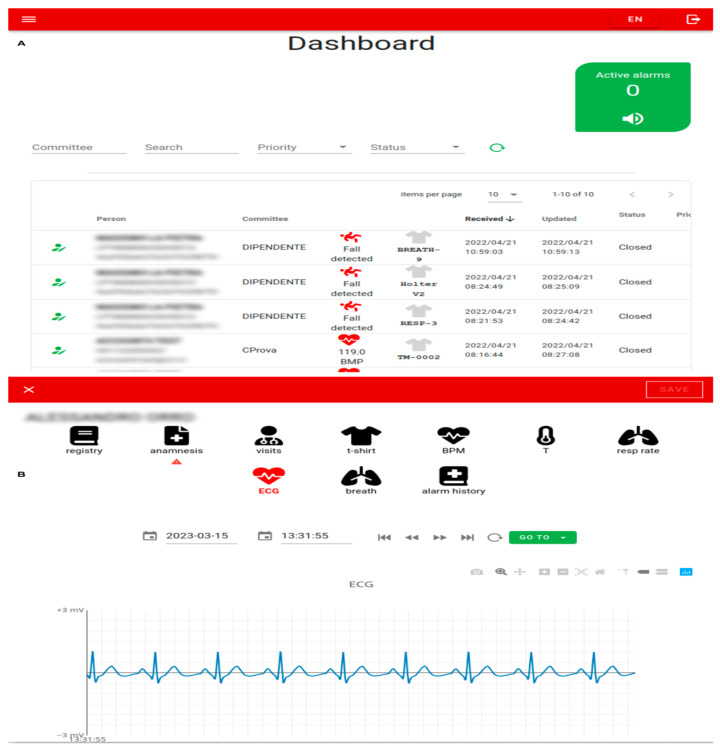
Some screenshots of the web Interface: (**A**) the dashboard of alarms and (**B**) details page for person related information: registry, health status, ECG (amplitude [mV] and time [s]) and monitored parameters during working shift.

The central component of the interface is the Dashboard page (Figure 8A), which displays the history of alarms received by the edge devices. The system manages four types of alarms: abnormal BPM values, abnormal breathing frequency, high temperature, and falls. Whenever the platform generates a new alarm, it is instantly captured by the web interface using websockets. Subsequently, it appears at the top of the list, triggering visual and audible alarms on the web page.

Based on the current permissions and roles (administrator, doctor, operator and volunteer), the user can view a set of clinical, physiological, and anamnestic parameters, as well as the outcome of medical visits. Additionally, it is possible to access data from IoT devices, such as ECG, respiratory trace, and temperature, with associated timestamps (Figure 8B).

The user can also navigate through temporal data using a menu that allows for immediate access to the raw trace associated with the moment an alarm is triggered or other critical points (for example, the maximum BPM value). This feature enables visual inspection of the specific data point linked to the alarm occurrence.

### 3.2. Workflows

**Figure 9:**
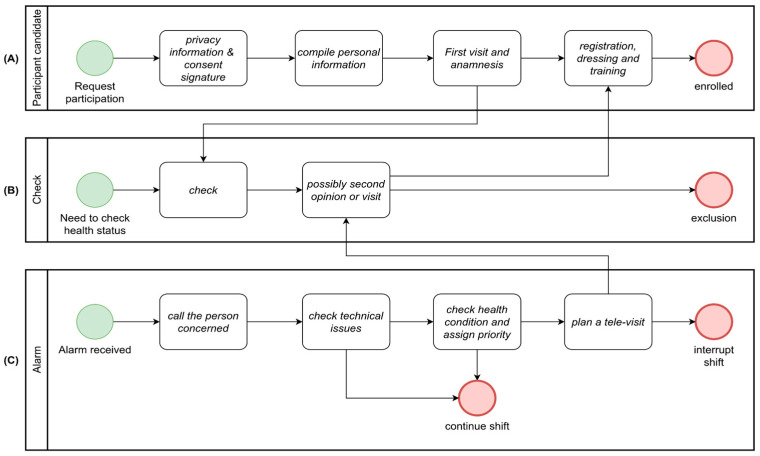
Some relevant workflows of the application. (**A**) participant onboarding, (**B**) checking of the health status, (**C**) alarm management.

The workflow of enrollment of participants is described in Figure 9A. They join after signing an informed consent for data processing based on European General Data Protection Regulation 2016/679 GDPR. Then, they undergo a medical examination to establish an updated and accurate clinical record, crucial for properly configuring device parameters and analyses. At this stage, a volunteer may be excluded from the study if deemed unfit (for instance, in cases of cardiac issues). In research study monitoring scenarios, identification data are removed through pseudo-anonymization methods. Additionally, there exists a provision to exclude patients from the ongoing analysis within the project at any juncture. This exclusion may occur if patients no longer meet the outlined requirements or choose to voluntarily withdraw their participation from the project. This mechanism ensures the integrity and accuracy of the ongoing analysis by allowing for the removal of data points that no longer align with the project’s criteria or are voluntarily retracted by the participants.

The alarm status follows the workflow depicted in Figure 9B. Once an event associated with an alarm is generated, it is immediately displayed on the desktop of the operators of the control center. An operator can promptly reach out to the individual concerned to verify if there have been any technical issues (such as connectivity problems or incorrect device usage) and provide assistance for resolution. If the event is a real health issue, the case is forwarded to the medical center with a priority (low, medium or high), where a doctor handles the situation, conducting a televisit and analysis of the data collected in the immediate time prior to the event. At this point, emergency services can be activated, and the worker’s activity can be promptly suspended.

Finally, Figure 9C displays the workflow for offline analysis of ECG signals, which can be performed through automated batch analysis to highlight both technical anomalies (such as excessive noise) and clinical irregularities. Medical staff can annotate the signal with messages visible to the entire team, facilitating a sequence of second opinions on the case, enabling a decision to schedule a visit.

### 3.3. Performance and Scalability

**Figure 10:**
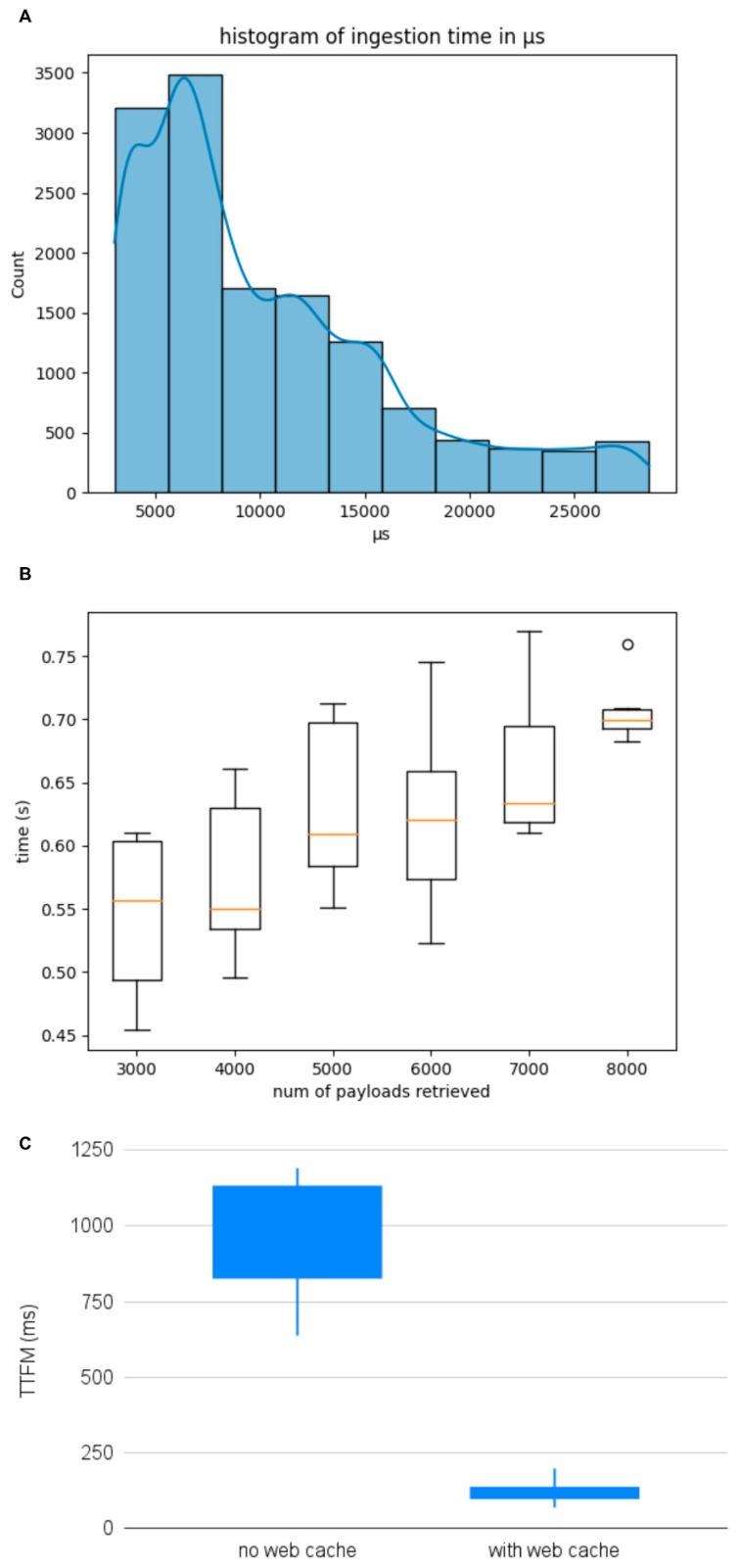
(**A**) histogram of ingestion time, (**B**) scalability analysis of read operations, (**C**) comparison of TTFB between normal case and use of web cache.

The response speed of a Cloud application is crucial for ensuring both a good user experience and optimal system performance and scalability. During the development and operation of the application, extensive testing was performed, and performance metrics were collected from the system logs and continuously analyzed to identify potential bottlenecks and optimize processing times. The ingestion time of raw data from IoT devices, in particular for ECG and respiratory traces, holds significant importance within this application. This timeframe encompasses the duration required for transmitting data from the devices to the backend through the MQTT protocol and the subsequent storage time. Figure 10A shows the histogram distribution of the ingestion time of a typical workflow session comprising 16,000 payloads of 1 s from 1000 devices. Simulations conducted in a controlled environment confirmed that the average ingestion time is 10 ± 0.4 µs, demonstrating the efficiency of the data ingestion pipeline.

In addition to these simulations, tests were conducted to assess the system’s performance under poor network conditions by deliberately interrupting mobile network access in a controlled manner. These interruptions simulated real-world scenarios where connectivity with the Cloud is limited or lost, leading to the smartphone’s cache filling up. This approach allowed us to ensure that the architecture could continue functioning even in the absence of a network connection for up to 15 h (depending on the local memory of the smartphone).

Figure 10B illustrates the scalability of the application when subjected to varying loads of read queries with different numbers of payloads. The tests simulate real-world conditions with increasing query loads to assess the system’s elastic response. As expected, due to the elastic capabilities of the Cloud infrastructure, the read time of raw data from the TimeStream DB scales linearly, maintaining performance under higher loads.

The final recorded metric is the Time to First Byte (TTFB), defined, from a web application perspective, as the duration from the initiation of the web request to the receipt of the first byte of the response. In this case, the time is also influenced by the computational capabilities of the client to generate the request (usually in JSON format) and the client networking. Values < 200 ms are considered good in modern applications. Performance tests comparing cache-enabled and non-cached scenarios showed improvements in TTFB, as seen in Figure 10C. Specifically, a simple web caching mechanism was implemented on a specific Cloud component to enhance user experience when navigating through the data stream, reducing TTFB by an average of 90% in cache-enabled scenarios. These experimental results confirm the advantages of the caching strategy in maintaining responsiveness, even under varying loads.

To address the limitations of traditional infrastructure, such as limited scalability and high maintenance costs, our Cloud-based solution leverages elastic scalability and automated backup capabilities, ensuring efficient data ingestion and response times. The results, including data on ingestion times and TTFB measurements, were obtained through rigorous testing under varying loads, demonstrating the advantages of Cloud architecture over traditional systems in terms of performance and reliability.

Overall, the system demonstrates good reliability due to the intrinsic resilience properties of the Cloud, including elasticity, redundancy, and fault tolerance. These features enable the application to adapt to varying loads and ensure continuous operation, even during peak usage times. On the other hand, the user’s smartphone represents a potential weakness when network disconnections occur; in these cases, it must accumulate data over time and subsequently send all collected data once it reconnects to the network. This aspect highlights the need for robust offline capabilities (internal memory and bandwidth) to maintain data integrity and prevent loss during connectivity issues.

## 4. Discussion

Continuous vital sign monitoring is pivotal in modern e-health, spanning hospital settings, home care, emergency management, and occupational safety. However, this task is laden with technical complexities, particularly in managing real-time data.

To address these challenges, we introduced a Cloud-based monitoring architecture designed for remote vital sign tracking for paramedics and medical personnel, leveraging low-cost, wearable IoT sensors. Continuous monitoring of vital signs, such as ECG and respiration, serves as a crucial tool in emergency management, reducing risks during rescue operations and ensuring worker safety.

This solution boasts inherent resilience and elasticity, effectively circumventing the limitations of traditional infrastructures while addressing the challenges posed by massive data volumes, network congestion, and the computational demands of IoT monitoring. Additionally, it orchestrates the management of essential health metrics during work sessions, providing real-time alerts to a central personnel management center. This implementation enables efficient tracking and immediate responses to critical events, exemplifying the practical application of wearable IoT technology in safeguarding the well-being of frontline workers.

We evaluated the system’s performance in its critical paths to assess scalability and robustness for larger case studies. In particular, caching systems and elastic features—such as resource adaptation based on load—have been integrated into all data paths of the Cloud infrastructure.

## 5. Conclusions

The proposed application exemplifies how technological innovation can be a game changer when tailored to the specific needs of particular user groups and operators, thus enhancing the adoption of modern approaches in healthcare and research.

The experience described proved invaluable in identifying limitations during the experimentation phase, particularly concerning wearability issues encountered during extensive movements. These challenges were addressed through a modeling process aimed at improving the fit of the textile garment.

In future work, we plan to further develop the platform in three directions: (1) integrating advanced textile technology to incorporate three ECG derivations for a fully compliant Holter clinical examination; (2) embedding a 4G and 5G modem to enable direct data transmission to the Cloud without relying on a smartphone; and (3) incorporating a set of new, more accurate cinematic sensors (IMU) to acquire motion detection data on the Cloud platform. These enhancements to the wearable device, alongside ongoing developments of the Cloud platform, will broaden the potential applications of the system in both clinical and non-clinical areas, such as workplace health protection and overall well-being.

This technological model is underpinned by a cooperative intent, facilitating multidisciplinary collaboration, even in challenging areas, like research on syndromic conditions, which are difficult to investigate without solutions such as YouCare and its monitoring platform. Recently, a study on RETT syndrome carriers and their caregivers was published [47], showcasing the success of real-time monitoring of biovital and environmental parameters, with YouCare playing an essential role in the research. Additionally, several studies focusing on the telemonitoring of adapted physical activity are currently underway, involving research groups in Neuromotor Science, Physiatry, Pharmacology, and Psychology. This illustrates how complex scenarios can serve as challenging ecosystems for the development of highly innovative solutions like WaidX [48], promoting the adoption of good health practices and the use of modern technologies in healthcare.

## Figures and Tables

**Table 1:** List and description of the components of the Cloud platform.

Component	Description
Web Worker	A stateless virtual node that manages http requests coming from the web clients.
Load Balancer	A component that distributes the http request over a set of web workers and manages the number of active nodes based on the overall load
Relational Database	The main database for operational activities including individual registry, device management and alert management.
TimeStream Database	NoSQL database for time stream data such as ECG, Respiratory Trace and heart Beats per minute (BPM).
Simple Queue Service (SQS)	A component that uses using message queue to handle asynchronous communication between microservices (Lambda functions, web workers and edge devices) ensuring decoupling of components
Lambda Function	A serverless and event-driven computing service used to manage asynchronous tasks such as the generation of alarms
IoT Core	The component that handles the connectivity between the Internet of Things (IoT) devices and the backend Cloud platform using a publish/subscribe communication based on (MQTT) protocol

**Table 2:** Anamnesis information is utilized as inclusion criteria for the experimentation conducted on a group of active workers engaged in emergency response operations within ambulances.

Category	Description
Familiar disease	Heart disease, diabetes, hypertension, neoplastic pathologies, sudden juvenile death
Physiological anamnesis	Weight, height, motor skill (full or reduced), heart rate, diastolic and systolic blood pressure
Lifestyle	Quantification of alcohol consumption and cigarette use
Pathological anamnesis	COVID-19, endocrine diseases, allergies, respiratory pathologies, liver or biliary diseases, neurological diseases, neoplastic pathologies, kidney or urinary tract disease
Interventions and hospitalizations	Type, age and duration of hospital admissions including related intervention related to surgical intervention

**Table 3:** Percentages of the ~4000 alerts generated by the platform during this study.

Alert Type	Percent
BPM	72%
Temperature	15%
Fall	13%
False positive	40%

## Data Availability

Data is contained within the article.
